# A systematic review of the performance of actigraphy in measuring sleep stages

**DOI:** 10.1111/jsr.14143

**Published:** 2024-02-21

**Authors:** Hang Yuan, Elizabeth A. Hill, Simon D. Kyle, Aiden Doherty

**Affiliations:** 1 Big Data Institute, University of Oxford, Oxford, UK; 2 Nuffield Department of Population Health, University of Oxford, Oxford, UK; 3 Sir Jules Thorn Sleep and Circadian Neuroscience Institute, University of Oxford, Oxford, UK

**Keywords:** accelerometer, actigraphy, polysomnography, sleep staging, systematic review, validity

## Abstract

The accuracy of actigraphy for sleep staging is assumed to be poor, but examination is limited. This systematic review aimed to assess the performance of actigraphy in sleep stage classification of adults. A systematic search was performed using MEDLINE, Web of Science, Google Scholar, and Embase databases. We identified eight studies that compared sleep architecture estimates between wrist-worn actigraphy and polysomnography. Large heterogeneity was found with respect to how sleep stages were grouped, and the choice of metrics used to evaluate performance. Quantitative synthesis was not possible, so we performed a narrative synthesis of the literature. From the limited number of studies, we found that actigraphy-based sleep staging had some ability to classify different sleep stages compared with polysomnography.

## 1 Introduction

Actigraphy has long been used to measure sleep–wake patterns in clinical and daily living environments. The broad adoption of actigraphy is driven by its relatively low cost, minimal user burden, and ease of continuous recordings over weeks (Sadeh, 2011). The American Academy of Sleep Medicine (AASM) published a meta-analysis on actigraphy for sleep detection in comparison with polysomnography (PSG) and concluded that actigraphy was suitable to assess total sleep time and sleep onset latency for clinical decision-making in adults with sleep disorders (Smith et al., 2018). Extensive evaluations of actigraphy have been done on its population validity (Conley et al., 2019; Schwab et al., 2018), ecological validity (Van de Water et al., 2011), and device validity (Chinoy et al., 2021; Ko et al., 2015) for sleep–wake classification.

Fundamentally, actigraphy and PSG measure different dimensions of sleep. PSG measures various physiological and movement signals, including brain activity (electroencephalography [EEG]), ocular movement (electro-oculography [EOG]), muscle functions (electromyography [EMG]) and heart rhythm (electrocardiography [ECG]), with the combination of EEG, EOG, and EMG forming the basis of objective determination of sleep stages in line with published international guidelines (Berry et al., 2020). In contrast, actigraphy measures peripheral limb movement alone, using activity as a proxy for wake and inactivity as a proxy for sleep, and by extension, has limited capacity to infer the stages of sleep due to the lack of physiological signals. Nonetheless, the use of actigraphy for sleep staging is relatively unknown. Understanding the performance limits of actigraphy-based sleep staging may open the door to investigate the association between sleep architecture and important clinical outcomes in large-scale biobank datasets, in which only actigraphy is available (Chen et al., 2011; Doherty et al., 2017).

The most relevant systematic review evaluated wearable sensing technologies, including actigraphy for sleep staging (Imtiaz, 2021). However, due to the broad coverage of the review, the depth of discussion related to each sensing modality was limited, and the review did not consider the technical parameters that could influence the algorithm performance for each device. Therefore, we set out to: (i) systematically evaluate the validity of sleep staging using actigraphy, and (ii) analyse the effect of technical parameters on sleep staging performance.

## 2 Methods

This review was conducted according to the Preferred Reporting Items for Systematic Reviews and Meta-Analyses (PRISMA) guidelines (Page et al., 2021). The protocol was registered with the International Prospective Register of Systematic Reviews (PROSPERO) following the PRISMA-P guidelines (PROSPERO 2021 CRD42021237456) prior to commencement.

### 2.1 Eligible studies

We included studies if they: (i) compared the sleep staging agreement between actigraphy and PSG for adults, (ii) reported agreement results on AASM sleep stages (wake (W)/non-rapid-eye-movement sleep (NREM) 1, 2, and 3 (N1, N2, and N3)/rapid-eye-movement sleep (R)) or performance on the composite of different sleep stages, and (iii) scored the PSG manually or used an automated PSG scoring first and then corrected the scoring by a human rater. Studies were included regardless of the PSG recording montage used, provided the scoring followed the AASM guidelines.

Our exclusion criteria were: (i) editorials, reviews, or commentaries; (ii) single-channel EEG as the reference when scoring sleep stages; (iii) studies that only reported the agreement performance for sleep and wakefulness; (iv) scoring using Rechtschaffen and Kales rules (Rechtschaffen, 1968). Table S1 lists the criteria for selecting eligible studies in detail.

### 2.2 Search strategies

The searches were performed using MEDLINE (Ovid), Web of Science, Google Scholar and Embase (Ovid) on July 25, 2023. We also included preprints that appeared in Google Scholar to mitigate publication bias. The exact search terms for each database are specified in Section S3. Our search strategy was adapted from previous work (Conley et al., 2019), which aimed to assess the agreement between actigraphy and PSG using parameters related to sleep and wake patterns. To focus more on the performance of sleep staging, we made two changes to the search terms: (i) included more research-grade and consumer-grade actigraphy to capture a broader set of studies using different wrist-worn accelerometers, and (ii) added ‘sleep stage’ to the search terms to make the search results relevant to sleep staging. In developing the search syntax for each database, we first determined the Ovid syntax for Embase and MEDLINE, then translated the search terms to other databases.

### 2.3 Study selection and data extraction

Literature search results were uploaded to Covidence (Covidence, Melbourne, Australia), an internet-based systematic review program that assists reviewers in streamlining the review process. Covidence automatically flagged any possible reference duplication. One reviewer (H.Y.) manually verified the duplication removal. H.Y. also conducted the literature search and abstract screening. A second reviewer (E.A.H.) independently verified that all the included abstracts meet the selection criteria. Full-text screening and data extraction were performed by both H.Y. and E.A.H. independently in duplicate. Initial reconciliation was undertaken by H.Y. and E.A.H., with a third author resolving any outstanding disagreement.

The key data fields for extraction included sleep staging outcomes, device specifications (device type, data modality and sampling frequencies), data acquisition environment, and population characteristics (sample size, age, sex, and existing comorbidities). Information related to the sleep staging algorithms, including algorithmic techniques and validation methods, was also extracted. When multiple algorithms were reported in a study, data were extracted only for the algorithm with the best performance. If different datasets were used for algorithm development and validation, then the performance of the validation dataset was extracted. Section S1 lists detailed data items. The data extraction form was piloted on a subset of studies during the screening process.

### 2.4 Data evaluation

The risk of bias was evaluated independently by H.Y. and E.A.H. using a modified Quality Assessment of Diagnostic Accuracy Studies-2 (QUADAS-2) tool (Whiting et al., 2011). Although QUADAS-2 has mainly been designed for primary diagnostic tools, the four key domains in QUADAS-2 are pertinent to the focus of the review: (i) patient selection, (ii) index test, (iii) reference standard, and (iv) flow and timing. The key domains reflect whether all the participants received the same reference standard and participant inclusions throughout the analyses. Domains 2 and 3 also reflected concerns around algorithmic validity. The following irrelevant signalling questions were removed from the original QUADAS-2: question 2 in patient selection, questions 1 and 2 in index test, question 2 in reference standard, and question 1 in flow and timing. Two additional questions were included to better assess algorithmic bias: question 1 in the index test and question 2 in the reference standard. The risk of bias is judged as ‘low’, ‘high’, and ‘unclear’. The risk of bias is judged as ‘low’ if answers to all signalling questions are ‘yes’; otherwise, the risk of bias is judged as ‘high’. When the information available does not permit answering the signalling questions, then the risk is judged as ‘unclear’. Table S4 entails the modified QUADAS-2 in full.

## 3 Results

**Figure 1:**
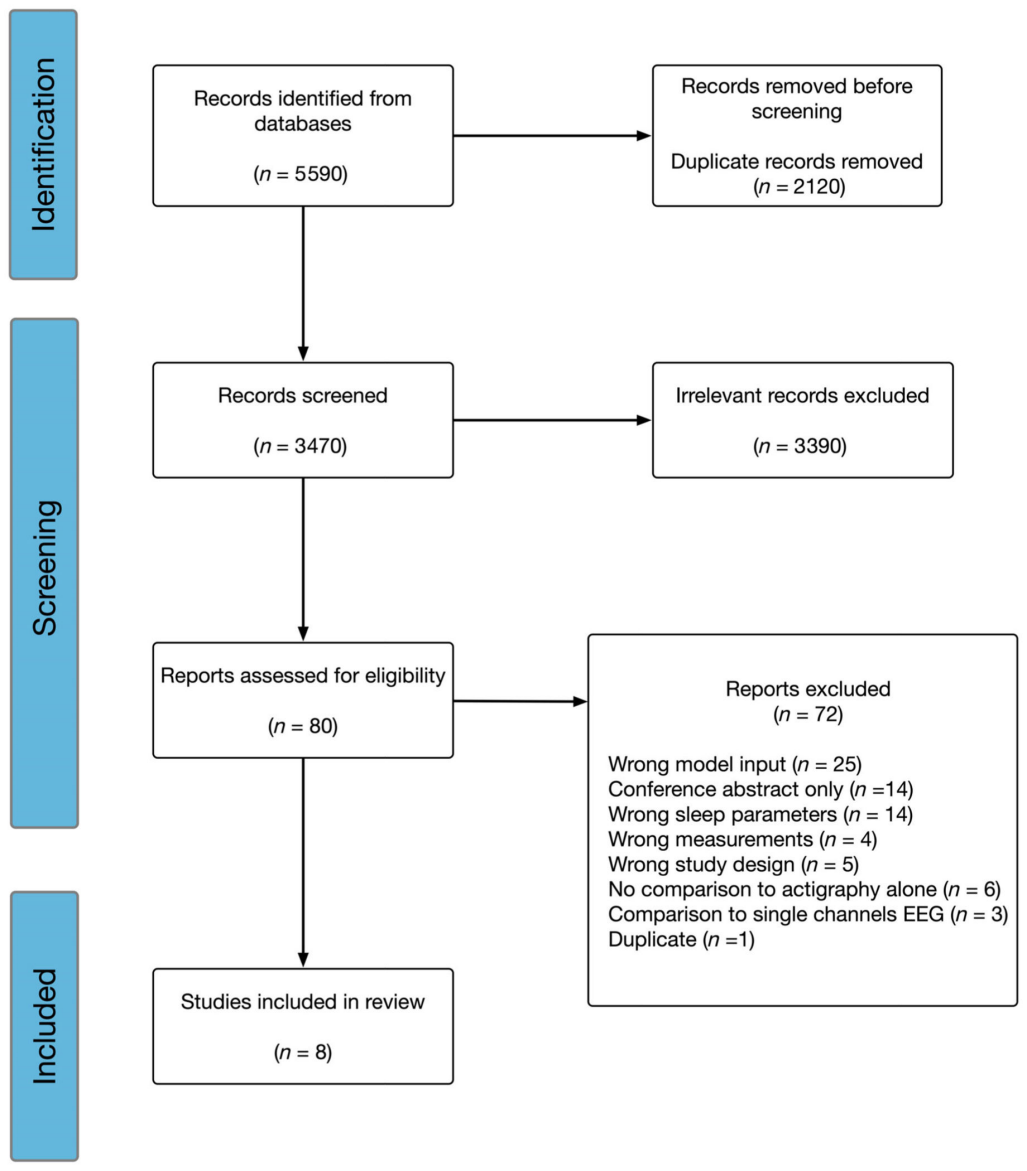
Preferred Reporting Items for Systematic Reviews and Meta-Analyses (PRISMA) flow diagram of studies screened and included to review the validity of actigraphy for the measurement of sleep stages. EEG, electroencephalography.

Our literature search identified 5590 records from the databases, 2120 of which were removed due to duplication by Covidence (Figure 1). Another 3390 records were removed after the abstract screening. Among the remaining 80 studies for full-text screening, wrong model input (*n* = 25), conference abstract only (*n* = 14), and wrong sleep parameters (*n* = 14) were the top three reasons for exclusion before data extraction. In total, eight studies met the inclusion criteria and were included in this review. Table S5 summarises the exact reasons for removal after the full-text screening.

### 3.1 Study populations

The population characteristics are listed in Table 1. The majority of studies used data from healthy participants (Boe et al., 2019; Devine et al., 2020; Mantua et al., 2016; Nguyen et al., 2021; Trevenen et al., 2019; Walch et al., 2019; Yuan et al., 2023), whereas one study assessed the sleep staging performance in participants with sleep disorders (Sundararajan et al., 2021). The number of participants included in each study tended to be small. All but two studies included <100 participants in their assessment (Sundararajan et al., 2021; Trevenen et al., 2019), both of which reported the sleep classification performance using cross-fold validation without external validation. Even though Yuan et al. (2023) also included >100 participants in their cross-fold cross-validation, the performance extracted is from external validation, which has only 52 participants.

### 3.2 Actigraphy devices

The full specifications of the actigraphy devices can be found in Table 1. Each study used a distinct type of device. Both research-grade and consumer-grade devices were used. There was no consensus on the device placement. Three studies placed the actigraphy on the non-dominant wrist; one study placed the device on the left wrist; two studies placed the device on both wrists, and two studies did not report the device placement. Both raw data and activity count were used for sleep staging inference: four studies used the raw data, two studies used activity count, and two studies did not report the data type used. The actigraphy sampling rate for different devices ranged from 30 to 62.5 Hz.

### 3.3 Sleep staging algorithms

Table 2 shows the algorithms used and the corresponding performance from each study. Seven studies reported algorithm details. Studies that used commercial-grade devices relied on the proprietary algorithms from the vendors that do not reveal the methods for sleep stage classification (Devine et al., 2020; Mantua et al., 2016). Heuristic-based methods were not used by any study. Statistical learning methods were the predominant method class, including logistic regression (Walch et al., 2019), Hidden Markov Model (Trevenen et al., 2019), and tree-based methods (Boe et al., 2019; Sundararajan et al., 2021). Deep learning techniques were used in some studies (Sundararajan et al., 2021; Yuan et al., 2023).

### 3.4 Performance metric and assessment

Several performance metrics were used to report the sleep stage outcome, including sensitivity, specificity, accuracy, F1 and the area under the receiver operating characteristic curve. Studies tended to report performance with different groupings of sleep stagings (Table 2). Hence, it is not possible to synthesise the evidence quantitatively. Only three studies reported the performance for all five AASM sleep stages (Sundararajan et al., 2021; Trevenen et al., 2019; Yuan et al., 2023). The highest average five-class performance was 42.5% in accuracy (Yuan et al., 2023), and 43.2% in F1 (Trevenen et al., 2019). As the number of classes decreases, the reported performance increases. For instance, an accuracy of 66.4% was reported in a three-class setting (W versus N1 + N2 versus N3 + R) (Nguyen et al., 2021). Studies using consumer-grade devices reported sleep staging performance by ‘light sleep’ and ‘deep sleep’ that were defined by the device vendors, making it impossible to compare sleep staging performance with other devices.

Method development studies used *N*-fold/leave-one-out cross-validation (Boe et al., 2019; Sundararajan et al., 2021; Trevenen et al., 2019; Walch et al., 2019; Yuan et al., 2023). Only one method development study reported the performance on datasets that the algorithm has not seen (Yuan et al., 2023). However, device validation studies used statistical testing or directly calculated the performance metrics (Devine et al., 2020; Mantua et al., 2016; Nguyen et al., 2021).

### 3.5 Risk of bias of included studies

Overall, the risk of bias for the included studies based on the modified QUADAS-2 was limited (Table 3). The greatest risk of bias came from the participant selection, just one study with low risk, four studies with high risk, and three studies with unclear risk. The next most common risk of bias was the index test having two studies with a high risk of bias, one with an unclear risk of bias, and five with a low risk of bias. Several studies aimed to develop sleep staging algorithms and thus included limited information about participant inclusion and did not consider appropriate participant inclusion for clinical use. Two studies that carried a high risk of bias for the index test domain used consumer-grade devices (Devine et al., 2020; Mantua et al., 2016) that relied on proprietary staging algorithms, which could introduce bias. All but one study carried a low risk of bias for the reference standard PSG.

## 4 Discussion

This review summarised the literature on the use of actigraphy in sleep staging. Eight studies were included, and only one study tested the validity of sleep staging in sleep patients. A large heterogeneity was found in the reporting of the performance in actigraphy-based sleep staging, especially around the sleep stage groupings and evaluation metrics, thus making it challenging to summarise existing evidence quantitatively. Nonetheless, based on the available studies, actigraphy-based sleep staging appears to have some ability to classify sleep stages.

Most of the evaluation of the actigraphy-based sleep staging has been conducted in young, healthy populations of limited size (*n* < 100). Only two of the included studies evaluated the sleep staging performance in >100 participants (Sundararajan et al., 2021; Trevenen et al., 2019). Sundararajan et al. (2021) was also the only study that evaluated the performance of actigraphy in patients with sleep disorders. Further evaluation of sleep staging performance in older adults and chronic conditions will help assess the clinical and scientific utility of actigraphy for sleep staging. However, the lack of open-access anonymous datasets requires researchers to collect their own datasets, which restricts the prospect of increasing the participant number and diversity for each study. Only one of the included studies used a dataset that was publicly available (Sundararajan et al., 2021). As a community, more effort should be put into making the data open access to allow for secondary data analysis.

The reporting of the sleep staging performance has been heterogeneous. Even though five sleep stages are defined by AASM, because of the difficulty in the sleep stage identification from actigraphy, several sleep stages are combined into one to improve the agreement with PSG. For example, Walch et al. (2019) conducted the evaluation in a three-class setting (W/N1 + N2/N3 + R). While sleep stage grouping will help to obtain better performance, five-class staging performance needs to be reported to aid the comparison across different studies. Furthermore, studies included used a range of evaluation metrics for multi-class classification. Future research will benefit from adopting existing reporting guidelines to increase performance comparison across studies (de Zambotti et al., 2022). In particular, researchers should report the staging performance at the individual five-stage level, which would enable better comparison between studies than the current practice of arbitrary groupings of the sleep stages. In particular, the sleep staging algorithm/device should be analysed in an epoch-by-epoch fashion to understand its performance in different sleep stages using a confusion matrix. If groupings are required, one should specify the grouping definition, scoring window length, and the AASM sleep stage equivalence for each stage in the new definition. In addition to reporting the sensitivity and specificity of each sleep stage, metrics robust to class imbalances should also be reported, including F1 and kappa scores. Finally, subject-wise performance should be reported so that others can assess inter-person variability.

Unlike sleep–wake classification for which heuristic-based methods are often used (Cole et al., 1992; Sadeh & Sharkey, 1994; Van Hees et al., 2015), all sleep staging methods reported here are based on statistical learning partly due to the difficulty of relying on pre-defined rules to infer sleep stages directly from wrist movement. Two studies leveraged recent advances in artificial intelligence, such as deep neural networks, but the model performance was limited (Sundararajan et al., 2021; Yuan et al., 2023). Data-driven methods like deep neural networks are particularly data-hungry and it would probably require thousands of nights worth of PSG data for deep neural networks-based methods to reach their potential.

Multi-modal methods for sleep staging predictions could help understand the value of each modality. A total of 25 references were excluded because of the wrong model input, and six studies were excluded because of no comparison to actigraphy alone. These groups of studies often used additional modalities such as heart rate or ECG to obtain better performance (Domingues et al., 2014; Haghayegh et al., 2020; Herscovici et al., 2007). Reporting modality-specific performance in a multi-modal setting will inform the best modalities to use.

The risk of bias assessment showed that the greatest risk of bias came from participant selection and the index test, actigraphy-based sleep staging in this case. For participant selection, all studies should report population demographics, recruitment approaches, and health conditions to allow others to assess the generalisability in different populations. The risk of bias for the index test mainly concerns the device validation studies, as the underlying algorithm is often not revealed. Sleep classification algorithms should be described, where, at the very least, the device hardware and the analysis software version should be reported.

### 4.1 Limitations

These results are likely to suffer from publication bias. It is possible that studies that could not classify sleep stages from actigraphy have not been published, particularly in individuals with chronic conditions for whom the classification will be more complex. Records were screened by a single reviewer in the initial abstract screening stage instead of two, as outlined in the study protocol, which may have inadvertently introduced some selection bias. However, all further steps in the review process were completed by two independent reviewers. Finally, this review did not consider the performance difference for studies with different validation methods due to the limited number of studies.

## 5 Conclusion

Even though the included studies carry a limited risk of bias indicating the validity of sleep staging from actigraphy measurement, the existing performance remains low. Current evidence does not support or refute the validity of sleep staging from actigraphy due to the heterogeneity in the outcome reporting among the included studies.

## Supplementary Material

Supplement

## Figures and Tables

**Table 1:** Study characteristics.

References	Participants *N*	Age, years Range/mean (SD)	Sex N(%)	Polysomnography		Actigraphy			
				Level and scoring guidelines	Setting	Placement	Data type	Sampling rate	Device
Nguyen et al., 2021	22	18–36	14 (63.6) F	Attended AASM 2012 [2] 30 nights in total	Lab.	Left wrist	NA	NA	Unknown
Walch et al., 2019	31 Healthy	29.4 (8.5)	21 (67.7) F	Attended AASM 2016 [4] 1 night per person	Lab.	NA	Raw data	50 Hz	Apple Watch (Series 2 and 3, Apple Inc., Cupertino, USA)
Trevenen et al., 2019	816 Healthy	22.1 (0.6)	413 (50.0) F	Attended AASM 2012 [2] 1 night per person	Lab.	Non-dominant wrist	Raw data	30 Hz	ActiGraph GT3X+ Monitor (Acti-Graph, Pensacola, USA)
Sundararajan et al., 2021	158 (70 sleep disorders and the rest healthy)	20–72	Not stated	Attended not stated	Lab.	NA	Raw data	50 Hz	Mostly GENEActiv (Activlnsights, Kimbolton, UK)
Mantua et al., 2016	40 Healthy	22.4 (4.9)	19 (47.5) F	Attended AASM 2007 [8] 1 night per person	Home	Both wrists	Activity count	NA	Misfit Shine (Misfit Wearables, San Francisco, USA)
Devine et al., 2020	8 Healthy	30.4 (3.2)	4 (50) F	Attended AASM 2012 [2] 3 nights per person	Lab.	Non-dominant wrist	NA	NA	Zulu watch (Institutes for Behaviour Resources, Baltimore, USA)
Boe et al., 2019	12 Healthy	27.4 (3.9)	5 (41.7) F	Attended AASM 2007 [8] 1 night per person	Lab.	Non-dominant wrist	Activity count	62.5 Hz	ActiWatch Spectrum (Philips Respironics; Murrysville, USA)
Yuan et al., 2023	52 Healthy	18–65	Not stated	Attended AASM 2007 [8] 1 night per person	Lab.	Both wrists	Rw data	30 Hz	Axivity (Axivity Ltd., Newcastle, UK)

Abbreviations: AASM, American Academy of Sleep Medicine; F, female; Lab., laboratory; NA, not available.

**Table 2:** Sleep staging algorithms and performance.

References	Algorithm	Validation	Sensitivity, %	Specificity, %	Accuracy, %	F1,%	AUROC
Nguyen et al., 2021	Not stated	Paired sample *t* test	W: 27.9 N1 + N2: 41.4 N3 + R: 65.8	W: 98.6 N1 + N2: 62.1 N3 + R: 51.6	Average: 66.4	NA	NA
Walch et al., 2019	Logistic regression	Leave one out cross-validation	NA	NA	W: 60 NREM: 50.6 R: 33.2	NA	NA
Trevenen et al., 2019	Hidden Markov model	Leave one out cross-validation	NA	NA	NA	W: 61.0 N1: 10.0 N2: 52.2 N3: 63.8 R: 6.4 Average: 43.2^a^	NA
Sundararajan et al., 2021	Random forest	*N*-fold cross-validation	NA	NA	W: 60.7 N1: 1.0 N2: 57.9 N3: 27.9 R: 17.5	W: 55.1 N1: 4.2 N2: 53.2 N3: 20.5 R: 12.3	NA
Mantua et al., 2016	Proprietary algorithm	Not stated	Light: 23.4 Deep: 69.6^b^	NA	NA	NA	NA
Devine et al., 2020	Proprietary algorithm	Not stated	Light: 10.7 Deep: 84.2	Light: 88.2 Deep: 30.0	Light: 51.7 Deep: 48.7^c^	NA	NA
Boe et al., 2019	Bagged trees	Leave one out cross-validation	NA	NA	NA	NA	W: 0.82 N1&N2: 0.58 N3: 0.64 R: 0.47^d^
Yuan et al., 2023	Deep neural network	*N*-fold cross-validation and external validation	NA	NA	W: 54.3 N1: 24.6 N2: 38.4 N3: 54.7 R: 40.5 Average: 42.5	NA	NA

Abbreviations: AUROC, area under the receiver operating characteristic curve; NA, not available; NREM, N1, N2, and N3, non-rapid-eye-movement sleep 1, 2, and 3; PSG, polysomnography; R, rapid-eye-movement sleep; W, Wake.

a The standard deviation for each class is: W (28.1), N1 (8.7), N2 (46.0), N (65.8), R (36.7).

b The average percentage errors are reported for the duration. Percentage errors: (Device duration – PSG duration)/PSG duration. Light: N1+N2; Deep sleep: N3+R.

c Each American Academy of Sleep Medicine PSG score is mapped from 0 (wake) to 3 (N3+R). Light sleep: any 2-min bins with an average score between 1 and 2.55. Deep sleep: any 2-min bin with an average score >2.5.

d The standard deviation for each class is: W (0.18), N1&N2 (0.05), N3 (0.1), R (0.09).

**Table 3:** Risk of bias using assessment Quality Assessment of Diagnostic Accuracy Studies-2 (QUADAS-2) tool.

Study	Risk of bias			Applicability concerns			
	Patient selection	Index test	Reference standard	Flow and timing	Patient selection	Index test	Reference standard
Nguyen et al., 2021	(−)	?	(−)	(−)	?	(−)	(+)
Walch et al., 2019	(−)	(+)	(+)	(+)	(+)	(+)	(+)
Trevenen et al., 2019	?	(+)	(+)	(+)	(+)	(+)	(+)
Sundararajan et al., 2021	?	(+)	(+)	?	?	(+)	(+)
Mantua et al., 2016	(−)	(−)	(+)	(+)	(+)	(+)	(+)
Devine et al., 2020	(−)	(−)	(+)	(+)	(+)	(+)	(+)
Boe et al., 2019	(+)	(+)	(+)	(+)	(+)	(+)	(+)
Yuan et al., 2023	?	(+)	(+)	?	?	(+)	(+)

*Note*: low risk, (+); high risk, (−); unknown risk, ?

## Data Availability

The data that supports the findings of this study are available in the supplementary material of this article.
