# Supplementary material for: A systematic review of the performance of actigraphy in measuring sleep stages
Source: J Sleep Res. Author manuscript; Available in PMC 2025 Oct 24. (PMC7618292; doi:10.1111/jsr.14143)
Supplement: Supplement [file EMS209481-supplement-Supplement.pdf]

Supplements

1. Data extraction item

- Authors, Reference number (text, number)
- Sample size (number)
- Funding sources (text)
- PSG type (text): at home recording or in a lab?
- PSG scoring rubric (text): R&K or AASM standard+year
- PSG channel/montage (text)
- PSG level if applicable: Level I/II (attended/unattended)
- Scoring method (sing-choice): manual, semi-automated, automated.
- Population type (text): age, population type (e.g. have sleep disorders or not),
- Recruitment approach (text)
- Device type/brand/mode
- Device placement
- Actigrpahy sampling rate
- Best performing algorithm name (text)
- Data source: activity count or raw accelerometery
- Sensitivity, specificity, accuracy and F1 for N1, N2, N3, combined NREM, R, and W.
- Balanced accuracy, kappa and F1 scores for all four sleep stages and W.

2. Eligibility criteria

Table 1: **Eligibility criteria**: accelerometer (ACC) and polysomnography (PSG).

	Inclusion	Exclusion
Literature language	English	Not English
Literature type	Empirical studies	Non-empirical works such as:<br>• Editorials<br>• Reviews<br>• Commentaries
Study design	Studies with parallel ACC and PSG.	Studies that have only ACC or PSG.
Populations	Adults age $\geq$ 18 years	Pediatric or adolescent populations with age $<$ 18 years
Measurements	Wrist-worn ACC and (PSG or EEG). Level I/II (attended/unattended) PSG.	• Studies that do not have ACC<br>• Studies that only has either ACC or (PSG or EEG)<br>• Studies that have both ACC and only single-channel EEG.
PSG scoring	Only consider manual or semi-automated (initially scored automatically and then corrected by a human rater)	fully-automated scoring
Sleep parameters	Should at least report one of the following<br>• N1<br>• N2<br>• N3<br>• REM (R)<br>• NREM<br>• Other metrics, being composed by any of the abovementioned measures, that are more specific than sleep.	Only reports sleep vs wake results or does not report any of the following<br>• N1<br>• N2<br>• N3<br>• REM (R)<br>• NREM<br>• Other metrics, being composed by any of the abovementioned measures, that are more specific than sleep.

3. Search terms

Table 2: Search terms and results (Ovid).

Term index	Search term	Result
1	exp Actigraphy/	9697
2	wearable*.mp.	-
3	actigraphy.ti,ab.	7386
4	actimetry.ti,ab.	276
5	Actiwatch.mp.	1051
6	Somnowatch.mp.	25
7	Sleepwatch.mp.	27
8	"WatchPAT 100".mp.	7
9	Action-W.mp.	14
10	"WP 100".mp.	13532
11	WatchPAT.mp.	8
12	Motionlogger.mp.	125
13	Actillume.mp.	124
14	wGT3X-BT.mp.	34
15	Gt3x.mp.	199
16	geneactiv.mp.	-
17	camntech.mp.	-
18	axivity.mp.	-
19	Fitbit.mp	-
20	"Apple watch".mp	-
21	acceleromet*.mp.	1355
22	accelometer*.mp.	25563
23	actigraph.ti,ab.	13
24	exp Wrist/	4314
25	wrist.ti,ab.	29128
26	exp Monitoring, Physiologic/	47840
27	exp "Sleep stage"/	6887
28	"Sleep classification".mp.	6887
29	26 or 27 or 28	39246
30	24 or 25	46096
31	29 and 30	54817
32	1 or 2 or 3 or 4 or 5 or 6 or 7 or 8 or 9 or 10 or 11 or 12 or 13 or 14 or 15 or 16 or 17 or 18 or 19 or 20 or 21 or 22 or 23 or 31	37168
33	exp Polysomnography/	39871
34	exp Electroencephalography/	117466
35	Polysomnography.ti,ab.	26779
36	Electroencephalography.ti,ab.	22198
37	33 or 34 or 35 or 36	161404
38	32 and 37	2535
39	limit 38 to (english language and (adult < 18 to 64 years> or aged < 65+ years>))	1173

Table 3: Search terms and results for Web of Science.

Term index	Search term	Result
1	TS=Actigraphy	7132
2	TS=wearable*	69303
3	TS= actimetry	241
4	TS=Actiwatch	418
5	TS=Somnowatch	9
6	TS=Sleepwatch	4
7	TS="WatchPAT 100"	0
8	TS="Action-W"	18
9	TS="WP 100"	10
10	TS=WatchPAT	68
11	TS=Motionlogger	41
12	TS=Actillume	23
13	TS=wGT3X-B	1
14	TS=Gt3x	1091
15	TS=geneactiv	189
16	TS=camntech.	33
17	TS=axivity	72
18	TS=Fitbit	1382
19	Fitbit.mp	-
20	TS="Apple watch"	352
21	TS=acceleromet*	53384
22	TS=accelometer*	9
23	TS=actigraph	3829
24	TS=Wrist	47472
25	TS=Monitoring Physiologic	5064
26	TS=sleep stages	16636
27	TS=sleep classification	7273
28	#24 OR #25 OR #26	27370
29	#28 AND #24	296
30	#1 or #2 or #3 or #4 or #5 or #6 or #7 or #8 or #9 or #10 or #11 or #12 or #13 or #14 or #15 or #16 or #17 or #18 or #19 or #20 or #21 or #22 or #22 or #23 or #29	126395
31	TS=Polysomnography	18441
32	TS=Electroencephalography	34342
33	#31 OR #32	2535
36	#30 and #33	2409

Embase and Medline. The above strategy is adopted from the strategy used in [1]. The search terms were used for both Embase and Medline. When performing the the search, the time limit for Embase was 1974 to present and the time limit for Medline was 1946 to present. The search terms and results are shown in Supplement Table 2

Web of Science. The search syntax was adopted from the Embase syntax. The search terms and results are shown in Supplement Table 3

Google Scholar. Stop at page 20 because no results are relevant on page 21. 10 links per page. (Actigraphy OR wearable OR actigraphy OR Actimetry OR Actiwatch OR Somnowatch OR Sleepwatch OR "WatchPAT 100" OR "Action-W" OR 'WP 100" OR WatchPAT OR Motionlogger OR Actillume OR "wGT3X-BT" OR Gt3x OR geneactiv OR camntech OR axivity OR Fitbit OR Apple watch OR accelerometer OR accelerometry OR actigraph OR (Wrist AND ((Monitoring OR Physiologic) OR Sleep stage OR Sleep classification)) AND (Polysomnography OR Electroencephalography)

We put all the references into EndNote to search for abstracts before uploading them to Covidence for further processing.

Table 4: Modified QUADAS-2 form. Bold text questions are newly added.

Domain	Patient selection	Index test	Reference standard	Flow and Timing
Description	Describe methods of patient selection: Describe included patients (prior testing, presentation, intended use of index test and setting):	Describe the index test and how it was conducted and interpreted:	Describe the reference standard and how it was conducted and interpreted:	Describe any patients who did not receive the index test(s) and/or reference standard or who were excluded from the 2x2 table (refer to flow diagram): Describe the time interval and any interventions between index test(s) and reference standard:
Signalling questions (yes/no/unclear)	1. Was a consecutive or random sample of patients enrolled? 2. Did the study avoid inappropriate exclusions?	**1. Was the index test obtained using a known algorithm?**	1. Is the reference standard likely to correctly classify the target condition? **2. Was the reference standard interpreted using all participants as the test sample?**	1. Did all patients receive a reference standard? 2. Did all patients receive the same reference standard? 3. Were all patients included in the analysis?
Risk of bias: High/low/unclear	Could the selection of patients have introduced bias?	Could the conduct or interpretation of the index test have introduced bias?	Could the reference standard, its conduct, or its interpretation have introduced bias?	Could the patient flow have introduced bias?
Concerns regarding applicability: High/low/unclear	Are there concerns that the included patients do not match the review question?	Are there concerns that the index test, its conduct, or interpretation differ from the review question?	Are there concerns that the target condition as defined by the reference standard does not match the review question?	

Excluded studies

Table 5: **Exclusion reasons for studies that might be eligible.** AASM: American Academy of Sleep Medicine.

Reference	Reason for exclusion
Haghayegh et al. [2]	Comparison to single channel EEG only
Darbyshire et al. [3]	Comparison to single channel EEG only
Toedebusch et al. [4]	Comparison to single channel EEG only
Menghini et al. [5]	Conference abstract only
Kumar and Dash [6]	Conference abstract only
Pollet et al. [7]	Conference abstract only
Kim, Shin, and Byun [8]	Conference abstract only
Kawhawage et al. [9]	Conference abstract only
Kahawage et al. [10]	Conference abstract only
Olsen et al. [11]	Conference abstract only
Samman et al. [12]	Conference abstract only
Collen and Lesage [13]	Conference abstract only
Chung et al. [14]	Conference abstract only
Alakuijala, Jokela, and Toppila [15]	Conference abstract only
Dietz-Terjung, Martin, and Schobel [16]	Conference abstract only
Baker et al. [17]	Conference abstract only
Allen et al. [18]	Conference abstract only
Devine et al. [19]	Duplicate
Domingues, Paiva, and Sanches [20]	No comparison to actigraphy alone
Alcantara et al. [21]	No comparison to actigraphy alone
Haghayegh et al. [22]	No comparison to actigraphy alone
Herscovici et al. [23]	No comparison to actigraphy alone
Willemen et al. [24]	No comparison to actigraphy alone
Hedner et al. [25]	No comparison to actigraphy alone

Kalkbrenner et al. [26]	Wrong measurements: no wrist actigraphy
Zaffaroni et al. [27]	Wrong measurements: no wrist actigraphy
Concheiro-Moscoso et al. [28]	Wrong measurements: no wrist actigraphy
Turetken, Van Zaen, and Delgado-Gonzalo [29]	Wrong measurements: use only hear rate
Haghayegh et al. [30]	Wrong model input: classification model input is not acceleration.
Moreno-Pino et al. [31]	Wrong model input: classification model input is not acceleration.
Liang and Chapa-Martell [32]	Wrong model input: classification model input is not acceleration.
Wulterkens et al. [33]	Wrong model input: classification model input is not acceleration.
Miller et al. [34]	Wrong model input: classification model input is not acceleration.
Miller et al. [35]	Wrong model input: classification model input is not acceleration.
Renevey et al. [36]	Wrong model input: classification model input is not acceleration.
Kuula and Pesonen [37]	Wrong model input: classification model input is not acceleration.
Muzet et al. [38]	Wrong model input: classification model input is not acceleration.
Zhang et al. [39]	Wrong model input: classification model input is not acceleration.
Kahawage et al. [40]	Wrong model input: classification model input is not acceleration.
Ribeiro et al. [41]	Wrong model input: classification model input is not acceleration.
Fujimoto, Ding, and Takahashi [42]	Wrong model input: classification model input is not acceleration.
Fonseca et al. [43]	Wrong model input: classification model input is not acceleration.
Fonseca et al. [44]	Wrong model input: classification model input is not acceleration.
Zambotti et al. [45]	Wrong model input: classification model input is not acceleration.

Zhang et al. [46]	Wrong model input: classification model input is not acceleration.
Schneider, Zhang, and Schwartz [47]	Wrong model input: classification model input is not acceleration.
Cook et al. [48]	Wrong model input: classification model input is not acceleration.
Bresler et al. [49]	Wrong model input: classification model input is not acceleration.
Choi et al. [50]	Wrong model input: classification model input is not acceleration.
Chinoy et al. [51]	Wrong model input: classification model input is not acceleration.
Willemen et al. [52]	Wrong model input: classification model input is not acceleration.
Stucky et al. [53]	Wrong model input: classification model input is not acceleration.
Onder et al. [54]	Wrong model input: classification model input is not acceleration.
Kanady et al. [55]	Wrong sleep parameters: no AASM sleep stage results were reported.
Kim et al. [56]	Wrong sleep parameters: no AASM sleep stage results were reported.
Barouni et al. [57]	Wrong sleep parameters: no AASM sleep stage results were reported.
Barouni et al. [58]	Wrong sleep parameters: no AASM sleep stage results were reported.
Beecroft et al. [59]	Wrong sleep parameters: no AASM sleep stage results were reported.
Choi et al. [60]	Wrong sleep parameters: no AASM sleep stage results were reported.
Cook, Prairie, and Plante [61]	Wrong sleep parameters: no AASM sleep stage results were reported.
Edinger et al. [62]	Wrong sleep parameters: no AASM sleep stage results were reported.
Danzig et al. [63]	Wrong sleep parameters: no AASM sleep stage results were reported.
Jean-Louis et al. [64]	Wrong sleep parameters: no AASM sleep stage results were reported.

Honma et al. [65]	Wrong sleep parameters: no AASM sleep stage results were reported.
Cook, Prairie, and Plante [66]	Wrong sleep parameters: no AASM sleep stage results were reported.
Mikulec et al. [67]	Wrong sleep parameters: no AASM sleep stage results were reported.
Aktaruzzaman et al. [68]	Wrong sleep parameters: no AASM sleep stage results were reported.
Shen et al. [69]	Wrong study design: no parallel actigraphy and polysomnography.
Levendowski et al. [70]	Wrong study design: no parallel actigraphy and polysomnography.
Bell et al. [71]	Wrong study design: no parallel actigraphy and polysomnography.
Barash et al. [72]	Wrong study design: no parallel actigraphy and polysomnography.
Alcantara et al. [73]	Wrong study design: no parallel actigraphy and polysomnography.

393

PRISMA 2020 Main Checklist

**TITLE**			
Title	1	Identify the report as a systematic review.	page 1
**ABSTRACT**			
Abstract	2	See the PRISMA 2020 for Abstracts checklist	
**INTRODUCTION**			
Rationale	3	Describe the rationale for the review in the context of existing knowledge.	page 2
Objectives	4	Provide an explicit statement of the objective(s) or question(s) the review addresses.	page 2
**METHODS**			
Eligibility criteria	5	Specify the inclusion and exclusion criteria for the review and how studies were grouped for the syntheses.	page 3; subsection: eligible studies
Information sources	6	Specify all databases, registers, websites, organisations, reference lists and other sources searched or consulted to identify studies. Specify the date when each source was last searched or consulted.	page 3; subsection: search strategies
Search strategy	7	Present the full search strategies for all databases, registers and websites, including any filters and limits used.	page 3; subsection: search strategies
Selection process	8	Specify the methods used to decide whether a study met the inclusion criteria of the review, including how many reviewers screened each record and each report retrieved, whether they worked independently, and if applicable, details of automation tools used in the process.	page 4; subsection: study selection and data extraction

(continued)

Data collection process	9	Specify the methods used to collect data from reports, including how many reviewers collected data from each report, whether they worked independently, any processes for obtaining or confirming data from study investigators, and if applicable, details of automation tools used in the process.	page 4; subsection: study selection and data extraction
Data items	10a	List and define all outcomes for which data were sought. Specify whether all results that were compatible with each outcome domain in each study were sought (e.g. for all measures, time points, analyses), and if not, the methods used to decide which results to collect.	page 4; subsection: study selection and data extraction
	10b	List and define all other variables for which data were sought (e.g. participant and intervention characteristics, funding sources). Describe any assumptions made about any missing or unclear information.	page 4; subsection: study selection and data extraction
Study risk of bias assessment	11	Specify the methods used to assess risk of bias in the included studies, including details of the tool(s) used, how many reviewers assessed each study and whether they worked independently, and if applicable, details of automation tools used in the process.	page 4; subsection: data evaluation
Effect measures	12	Specify for each outcome the effect measure(s) (e.g. risk ratio, mean difference) used in the synthesis or presentation of results.	not applicable: no quantitative synthesis done
Synthesis methods	13a	Describe the processes used to decide which studies were eligible for each synthesis (e.g. tabulating the study intervention characteristics and comparing against the planned groups for each synthesis (item 5)).	not applicable: no quantitative synthesis done

395

(continued)

	13b	Describe any methods required to prepare the data for presentation or synthesis, such as handling of missing summary statistics, or data conversions.	not applicable: no quantitative synthesis done
	13c	Describe any methods used to tabulate or visually display results of individual studies and syntheses.	not applicable: no quantitative synthesis done
	13d	Describe any methods used to synthesize results and provide a rationale for the choice(s). If meta-analysis was performed, describe the model(s), method(s) to identify the presence and extent of statistical heterogeneity, and software package(s) used.	not applicable: no quantitative synthesis done
	13e	Describe any methods used to explore possible causes of heterogeneity among study results (e.g. subgroup analysis, meta-regression).	not applicable: no quantitative synthesis done
	13f	Describe any sensitivity analyses conducted to assess robustness of the synthesized results.	not applicable: no quantitative synthesis done
Reporting bias assessment	14	Describe any methods used to assess risk of bias due to missing results in a synthesis (arising from reporting biases).	not applicable: no quantitative synthesis done
Certainty assessment	15	Describe any methods used to assess certainty (or confidence) in the body of evidence for an outcome.	not applicable: no quantitative synthesis done
**RESULTS**			
Study selection	16a	Describe the results of the search and selection process, from the number of records identified in the search to the number of studies included in the review, ideally using a flow diagram.	page 4; last paragraph
	16b	Cite studies that might appear to meet the inclusion criteria, but which were excluded, and explain why they were excluded.	page 4; last paragraph
Study characteristics	17	Cite each included study and present its characteristics.	figure 1

(continued)

Risk of bias in studies	18	Present assessments of risk of bias for each included study.	table 3
Results of individual studies	19	For all outcomes, present, for each study: (a) summary statistics for each group (where appropriate) and (b) an effect estimate and its precision (e.g. confidence/credible interval), ideally using structured tables or plots.	table 2
Results of syntheses	20a	For each synthesis, briefly summarise the characteristics and risk of bias among contributing studies.	page 6; subsection: risk of bias of included studies
	20b	Present results of all statistical syntheses conducted. If meta-analysis was done, present for each the summary estimate and its precision (e.g. confidence/credible interval) and measures of statistical heterogeneity. If comparing groups, describe the direction of the effect.	not applicable: no statistic syntheses done
	20c	Present results of all investigations of possible causes of heterogeneity among study results.	not applicable: no statistic syntheses done
	20d	Present results of all sensitivity analyses conducted to assess the robustness of the synthesized results.	not applicable: no statistic syntheses done
Reporting biases	21	Present assessments of risk of bias due to missing results (arising from reporting biases) for each synthesis assessed.	page 12; subsection: limitations
Certainty of evidence	22	Present assessments of certainty (or confidence) in the body of evidence for each outcome assessed.	not applicable for diagnostic test review but for treatment
**DISCUSSION**			
Discussion	23a	Provide a general interpretation of the results in the context of other evidence.	page 11; discussion 1st paragraph
	23b	Discuss any limitations of the evidence included in the review.	page 12; subsection: limitations
	23c	Discuss any limitations of the review processes used.	page 12; subsection: limitations

(continued)

	23d	Discuss implications of the results for practice, policy, and future research.	page 12; 3rd paragraph
**OTHER INFORMATION**			
Registration and protocol	24a	Provide registration information for the review, including register name and registration number, or state that the review was not registered.	page 3
	24b	Indicate where the review protocol can be accessed, or state that a protocol was not prepared.	page 3
	24c	Describe and explain any amendments to information provided at registration or in the protocol.	page 12; section: limitations
Support	25	Describe sources of financial or non-financial support for the review, and the role of the funders or sponsors in the review.	page 13; section: acknowledgements
Competing interests	26	Declare any competing interests of review authors.	page 13; section: conflicts of interests
Availability of data, code and other materials	27	Report which of the following are publicly available and where they can be found: template data collection forms; data extracted from included studies; data used for all analyses; analytic code; any other materials used in the review.	page 13; sections: data and materials sharing

PRIMSA Abstract Checklist

**TITLE**			
Title	1	Identify the report as a systematic review.	Yes
**BACKGROUND**			
Objectives	2	Provide an explicit statement of the main objective(s) or question(s) the review addresses.	Yes
**METHODS**			
Eligibility criteria	3	Specify the inclusion and exclusion criteria for the review.	Yes
Information sources	4	Specify the information sources (e.g. databases, registers) used to identify studies and the date when each was last searched.	Yes
Risk of bias	5	Specify the methods used to assess risk of bias in the included studies.	No
Synthesis of results	6	Specify the methods used to present and synthesize results.	Yes
**RESULTS**			
Included studies	7	Give the total number of included studies and participants and summarise relevant characteristics of studies.	Yes
Synthesis of results	8	Present results for main outcomes, preferably indicating the number of included studies and participants for each. If meta-analysis was done, report the summary estimate and confidence/credible interval. If comparing groups, indicate the direction of the effect (i.e. which group is favoured).	Yes
**DISCUSSION**			
Limitations of evidence	9	Provide a brief summary of the limitations of the evidence included in the review (e.g. study risk of bias, inconsistency and imprecision).	No
Interpretation	10	Provide a general interpretation of the results and important implications.	Yes
**OTHER**			
Funding	11	Specify the primary source of funding for the review.	No
Registration	12	Provide the register name and registration number.	Yes

From: Page MJ, McKenzie JE, Bossuyt PM, Boutron I, Hoffmann TC, Mulrow CD, et al. The PRISMA 2020 statement: an updated guideline for reporting systematic reviews. MetaArXiv. 2020, September 14. DOI: 10.31222/osf.io/v7gm2. For more information, visit: www.prisma-statement.org

References

[1] Samantha Conley et al. “Agreement between actigraphic and polysomnographic measures of sleep in adults with and without chronic conditions: a systematic review and meta-analysis”. In: *Sleep Medicine Reviews* 46 (2019), pp. 151–160.

[2] Shahab Haghayegh et al. “Performance assessment of new-generation Fitbit technology in deriving sleep parameters and stages”. In: *Chronobiology international* 37.1 (2020), pp. 47–59.

[3] Julie L Darbyshire et al. “Measuring sleep in the intensive care unit: Electroencephalogram, actigraphy, or questionnaire?” In: *Journal of the Intensive Care Society* 21.1 PG - 22-27 (2020), pp. 22–27. DOI: https://dx.doi.org/10.1177/1751143718816910.

[4] Cristina D Toedebusch et al. “Multi-Modal Home Sleep Monitoring in Older Adults”. In: *Journal of visualized experiments : JoVE* 143 PG - (2019). DOI: https://dx.doi.org/10.3791/58823.

[5] L Menghini et al. “Accuracy of a commercial wearable in detecting sleep stages compared to polysomnography in adults: Considering sleep classification methods and effects of evening alcohol consumption”. In: *Sleep* 43.SUPPL 1 PG - A456-A457 (2020), A456–A457. DOI: http://dx.doi.org/10.1093/sleep/zsaa056.1187.

[6] S Kumar and S S Dash. “Nonparametric approach for sleep stage classification using cardiorespiratory and movement features”. In: *Journal of sleep research* 27.Supplement 1 PG - 231 (2018), p. 231. DOI: http://dx.doi.org/10.1111/jsr.12751.

[7] E P Pollet et al. “Activity trackers as a tool in sleep research: determining discrepancies in trackers vs. psg”. In: *Sleep* 43.SUPPL 1 PG - A460-A461 (2020), A460–A461. DOI: http://dx.doi.org/10.1093/sleep/zsaa056.1199.

[8] D Kim, W Shin, and J Byun. “Utility of fitbit charge 2 for sleep monitoring in patients with obstructive sleep apnea”. In: *Sleep* 43.SUPPL 1 PG - A458 (2020), A458. DOI: http://dx.doi.org/10.1093/sleep/zsaa056.1191.

[9] P Kawhawage et al. “Validation of consumer and research-grade activity trackers in Insomnia Disorder I: In-lab validation against polysomnography”. In: *Journal of sleep research* 28.SUPPL 1 PG - (2019). DOI: http://dx.doi.org/10.1111/jsr.12913.

[10] P Kahawage et al. "Validation of a consumer activity tracker against polysomnography and actigraphy in insomnia". In: *Sleep* 42.Supplement 1 PG - A128 (2019), A128. DOI: http://dx.doi.org/10.1093/sleep/zsz067.312.

[11] M Olsen et al. "Sleep stage prediction and sleep disordered breathing detection using raw actigraphy and photoplethysmography from wearable consumer device". In: *Sleep* 43.SUPPL 1 PG - A461-A462 (2020), A461–A462. DOI: http://dx.doi.org/10.1093/sleep/zsaa056.1202.

[12] H Samman et al. "Utility of actigraphy and polysomnography prior to conducting the adult multiple sleep latency test". In: *Sleep medicine* 64.Supplement 1 PG - S242 (2019), S242. DOI: http://dx.doi.org/10.1016/j.sleep.2019.11.677.

[13] J Collen and S Lesage. "The accuracy of actigraphy in the evaluation of patients with hypersomnia". In: *Sleep* 36.SUPPL. 1 PG - A251-A252 (2013), A251–A252.

[14] J Chung et al. "Racial/ethnic differences in actigraphy, questionnaire, and polysomnographymeasured indicators of sleep health and sleep quality: the multi-ethnic study of atherosclerosis". In: *Sleep* 43.SUPPL 1 PG - A138-A139 (2020), A138–A139. DOI: http://dx.doi.org/10.1093/sleep/zsaa056.360.

[15] A Alakuijala, T Jokela, and J Toppila. "Concomitant actigraphy and polysomnography at home in different sleep disorders". In: *Journal of sleep research* 27.Supplement 1 PG - 136 (2018), p. 136. DOI: http://dx.doi.org/10.1111/jsr.12751.

[16] S Dietz-Terjung, A Martin, and C Schobel. "A novel algorithm for the estimation of sleep states based on breathing and movement". In: *Sleep* 43.SUPPL 1 PG - A170 (2020), A170. DOI: http://dx.doi.org/10.1093/sleep/zsaa056.442.

[17] F Baker et al. "Validation of a multi-sensory commercially available wristband in measuring sleep composition against polysomnography". In: *Sleep medicine* 40.Supplement 1 PG - e21-e22 (2017), e21–e22.

[18] S Allen et al. "Identification of objective and subjective markers of sleep health". In: *Sleep* 41.Supplement 1 PG - A125 (2018), A125.

[19] Jaime K Devine et al. "Validation of Zulu Watch against Polysomnography and Actigraphy for On-Wrist Sleep-Wake Determination and Sleep-Depth Estimation". In: *Sensors (Basel, Switzerland)* 21.1 PG - (2020). DOI: https://dx.doi.org/10.3390/s21010076.

[20] Alexandre Domingues, Teresa Paiva, and J Miguel Sanches. “Hypnogram and sleep parameter computation from activity and cardiovascular data”. In: *IEEE transactions on bio-medical engineering* 61.6 PG - 1711-9 (2014), pp. 1711–1719. DOI: https://dx.doi.org/10.1109/TBME.2014.2301462.

[21] C Alcantara et al. “Sleep disturbances and depression in the multi-ethnic study of atherosclerosis”. In: *Sleep* 38.SUPPL. 1 PG - A328 (2015), A328.

[22] Shahab Haghayegh et al. “Deep Neural Network Sleep Scoring Using Combined Motion and Heart Rate Variability Data”. In: *Sensors (Basel, Switzerland)* 21.1 PG - (2020). DOI: https://dx.doi.org/10.3390/s21010025.

[23] Sarah Herscovici et al. “Detecting REM sleep from the finger: an automatic REM sleep algorithm based on peripheral arterial tone (PAT) and actigraphy”. In: *Physiological measurement* 28.2 PG - 129-40 (2007), pp. 129–140.

[24] Tim Willemen et al. “Automatic sleep stage classification based on easy to register signals as a validation tool for ergonomic steering in smart bedding systems”. In: *WORK-A JOURNAL OF PREVENTION ASSESSMENT & REHABILITATION* 41.1 PG - 1985-1989 (2012), pp. 1985–1989. DOI: 10.3233/WOR-2012-0419-1985.

[25] Jan Hedner et al. “Sleep staging based on autonomic signals: a multi-center validation study”. In: *Journal of clinical sleep medicine : JCSM : official publication of the American Academy of Sleep Medicine* 7.3 PG - 301-6 (2011), pp. 301–306. DOI: https://dx.doi.org/10.5664/JCSM.1078.

[26] Christoph Kalkbrenner et al. “Automated sleep stage classification based on tracheal body sound and actigraphy”. In: *German medical science : GMS e-journal* 17.PG - Doc02 (2019), Doc02. DOI: https://dx.doi.org/10.3205/000268.

[27] A Zaffaroni et al. “Non-Contact Estimation of Sleep Staging”. In: 65.PG - 77-80 (2018), pp. 77–80. DOI: 10.1007/978-981-10-5122-7_20.

[28] Patricia Concheiro-Moscoso et al. “Study Protocol on the Validation of the Quality of Sleep Data from Xiaomi Domestic Wristbands”. In: *INTERNATIONAL JOURNAL OF ENVIRONMENTAL RESEARCH AND PUBLIC HEALTH* 18.3 PG - (2021). DOI: 10.3390/ijerph18031106.

[29] Engin Turetken, Jerome Van Zaen, and Ricard Delgado-Gonzalo. “Embedded Deep Learning for Sleep Staging”. In: PG - 95-96 (2019), pp. 95–96. DOI: 10.1109/SDS.2019.00005.

[30] Shahab Haghayegh et al. "Deep Neural Network Sleep Scoring Using Combined Motion and Heart Rate Variability Data". In: *Sensors* 21.1 PG - (2021). DOI: 10.3390/s21010025.

[31] Fernando Moreno-Pino et al. "Validation of Fitbit Charge 2 and Fitbit Alta HR Against Polysomnography for Assessing Sleep in Adults With Obstructive Sleep Apnea". In: *Journal of clinical sleep medicine : JCSM : official publication of the American Academy of Sleep Medicine* 15.11 PG - 1645-1653 (2019), pp. 1645–1653. DOI: https://dx.doi.org/10.5664/jcsm.8032.

[32] Zilu Liang and Mario Alberto Chapa-Martell. "Accuracy of Fitbit Wristbands in Measuring Sleep Stage Transitions and the Effect of User-Specific Factors". In: *JMIR mHealth and uHealth* 7.6 PG - e13384 (2019), e13384. DOI: https://dx.doi.org/10.2196/13384.

[33] Bernice M Wulterkens et al. "It is all in the wrist: wearable sleep staging in a clinical population versus reference polysomnography". In: *Nature and Science of Sleep* 13.PG - 885 (2021), p. 885.

[34] Dean J Miller et al. "A validation study of the WHOOP strap against polysomnography to assess sleep". In: *JOURNAL OF SPORTS SCIENCES* 38.22 PG - 2631-2636 (2020), pp. 2631–2636. DOI: https://dx.doi.org/10.1080/02640414.2020.1797448.

[35] Dean J Miller et al. "A Validation Study of a Commercial Wearable Device to Automatically Detect and Estimate Sleep". In: *Biosensors* 11.6 PG - 185 (2021), p. 185.

[36] Ph Renevey et al. "Optical wrist-worn device for sleep monitoring". In: 65.PG - 615-618 (2018), pp. 615–618. DOI: 10.1007/978-981-10-5122-7_154.

[37] L Kuula and A K Pesonen. "Heart Rate Variability and Firstbeat Method for Detecting Sleep Stages in Healthy Young Adults: Feasibility Study". In: *JMIR mHealth and uHealth* 9.2 PG - e24704 (2021), e24704. DOI: http://dx.doi.org/10.2196/24704.

[38] A Muzet et al. "Assessing sleep architecture and continuity measures through the analysis of heart rate and wrist movement recordings in healthy subjects: Comparison with results based on polysomnography". In: *Sleep medicine* 21.PG - 47-56 (2016), pp. 47–56. DOI: http://dx.doi.org/10.1016/j.sleep.2016.01.015.

[39] Yuezhou Zhang et al. "Sleep Stage Classification Using Bidirectional LSTM in Wearable Multi-sensor Systems". In: PG - 443-448 (2019), pp. 443–448.

[40] Piyumi Kahawage et al. “Validity, potential clinical utility, and comparison of consumer and research-grade activity trackers in Insomnia Disorder I: In-lab validation against polysomnography”. In: *Journal of sleep research* 29.1 PG - e12931 (2020), e12931. DOI: `https://dx.doi.org/10.1111/jsr.12931`.

[41] A Ribeiro et al. “Evaluation of the Watch-PAT apparatus as a sleep-staging tool”. In: *Sleep* 40.Supplement 1 PG - A120-A121 (2017), A120–A121.

[42] K Fujimoto, Y Ding, and E Takahashi. “Sleep stage detection using a wristwatch-type physiological sensing device”. In: *SLEEP AND BIOLOGICAL RHYTHMS* 16.4 PG - 449-456 (2018), pp. 449–456. DOI: `http://dx.doi.org/10.1007/s41105-018-0175-5`.

[43] P Fonseca et al. “Validation of photoplethysmography-based sleep staging compared with polysomnography in healthy middle-aged adults”. In: *Sleep* 40.7 PG - 097 (2017), p. 97. DOI: `http://dx.doi.org/10.1093/sleep/zsx097`.

[44] Pedro Fonseca et al. “Automatic sleep staging using heart rate variability, body movements, and recurrent neural networks in a sleep disordered population”. In: *Sleep* 43.9 PG - (2020). DOI: `https://dx.doi.org/10.1093/sleep/zsaa048`.

[45] Massimiliano de Zambotti et al. “A validation study of Fitbit Charge 2 TM compared with polysomnography in adults”. In: *Chronobiology international* 35.4 PG - 465-476 (2018), pp. 465–476. DOI: `https://dx.doi.org/10.1080/07420528.2017.1413578`.

[46] Xin Zhang et al. “Sleep stage classification based on multi-level feature learning and recurrent neural networks via wearable device”. In: *Computers in biology and medicine* 103.PG - 71-81 (2018), pp. 71–81. DOI: `https://dx.doi.org/10.1016/j.compbiomed.2018.10.010`.

[47] H Schneider, Z Zhang, and A Schwartz. “A comparison of automated and manual sleep staging and respiratory event recognition in a portable sleep diagnostic device with in-lab sleep study”. In: *EUROPEAN RESPIRATORY JOURNAL* 56.Supplement 64 PG - (2020). DOI: `http://dx.doi.org/10.1183/13993003.congress-2020.2100`.

[48] Jesse D Cook et al. “Ability of the Fitbit Alta HR to quantify and classify sleep in patients with suspected central disorders of hypersomnolence: A comparison against polysomnography”. In: *Journal of sleep research* 28.4 PG - e12789 (2019), e12789. DOI: `https://dx.doi.org/10.1111/jsr.12789`.

[49] Ma'ayan Bresler et al. "Differentiating between light and deep sleep stages using an ambulatory device based on peripheral arterial tonometry". In: *Physiological measurement* 29.5 PG - 571-84 (2008), pp. 571–584. DOI: https://dx.doi.org/10.1088/0967-3334/29/5/004.

[50] Ji Ho Choi et al. "Validation study of portable device for the diagnosis of obstructive sleep apnea according to the new AASM scoring criteria: Watch-PAT 100". In: *Acta oto-laryngologica* 130.7 PG - 838-43 (2010), pp. 838–843. DOI: https://dx.doi.org/10.3109/00016480903431139.

[51] Evan D Chinoy et al. "Performance of Seven Consumer Sleep-Tracking Devices Compared with Polysomnography". In: *Sleep* PG - (2020). DOI: https://dx.doi.org/10.1093/sleep/zsaa291.

[52] T Willemen et al. "An Evaluation of Cardiorespiratory and Movement Features With Respect to Sleep-Stage Classification". In: *IEEE JOURNAL OF BIOMEDICAL AND HEALTH INFORMATICS* 18.2 PG - 661-669 (2014), pp. 661–669. DOI: 10.1109/JBHI.2013.2276083.

[53] Benjamin Stucky et al. "Validation of Fitbit Charge 2 Sleep and Heart Rate Estimates Against Polysomnographic Measures in Shift Workers: Naturalistic Study". In: *J Med Internet Res* 23.10 PG - e26476 (2021), e26476. DOI: 10.2196/26476.

[54] Nilgun Surmen Onder et al. "Watch peripheral arterial tonometry in the diagnosis of obstructive sleep apnea: influence of aging". In: *The Laryngoscope* 122.6 PG - 1409-14 (2012), pp. 1409–1414. DOI: https://dx.doi.org/10.1002/lary.23233.

[55] Jennifer C Kanady et al. "Validation of sleep measurement in a multisensor consumer grade wearable device in healthy young adults". In: *Journal of clinical sleep medicine : JCSM : official publication of the American Academy of Sleep Medicine* 16.6 PG - 917-924 (2020), pp. 917–924. DOI: https://dx.doi.org/10.5664/jcsm.8362.

[56] M J Kim et al. "Comparison of three actigraphic algorithms used to evaluate sleep in patients with obstructive sleep apnea". In: *Sleep and Breathing* 17.1 PG - 297-304 (2013), pp. 297–304. DOI: http://dx.doi.org/10.1007/s11325-012-0689-z.

[57] Amna Barouni et al. "Ambulatory sleep scoring using accelerometers-distinguishing between nonwear and sleep/wake states". In: *PEERJ* 8.PG - e8284 (2020), e8284. DOI: https://dx.doi.org/10.7717/peerj.8284.

[58] A Barouni et al. “Ambulatory sleep scoring using accelerometers-distinguishing between nonwear and sleep/wake states”. In: *PEERJ* 2020.1 PG - 8284 (2020), p. 8284. DOI: http://dx.doi.org/10.7717/peerj.8284.

[59] Jaime M Beecroft et al. “Sleep monitoring in the intensive care unit: comparison of nurse assessment, actigraphy and polysomnography”. In: *INTENSIVE CARE MEDICINE* 34.11 PG - 2076-83 (2008), pp. 2076–2083. DOI: https://dx.doi.org/10.1007/s00134-008-1180-y.

[60] Su Jung Choi et al. “Discordant sleep parameters among actigraphy, polysomnography, and perceived sleep in patients with sleep-disordered breathing in comparison with patients with chronic insomnia disorder”. In: *Sleep & breathing = Schlaf & Atmung* 21.4 PG - 837-843 (2017), pp. 837–843. DOI: https://dx.doi.org/10.1007/s11325-017-1514-5.

[61] Jesse D Cook, Michael L Prairie, and David T Plante. “Utility of the Fitbit Flex to evaluate sleep in major depressive disorder: A comparison against polysomnography and wrist-worn actigraphy”. In: *Journal of affective disorders* 217.PG - 299-305 (2017), pp. 299–305. DOI: https://dx.doi.org/10.1016/j.jad.2017.04.030.

[62] Jack D Edinger et al. “A pilot study of inexpensive sleep-assessment devices”. In: *BEHAVIORAL SLEEP MEDICINE* 2.1 PG - 41-9 (2004), pp. 41–49.

[63] Rachel Danzig et al. “The wrist is not the brain: Estimation of sleep by clinical and consumer wearable actigraphy devices is impacted by multiple patient- and device-specific factors”. In: *Journal of sleep research* 29.1 PG - e12926 (2020), e12926. DOI: https://dx.doi.org/10.1111/jsr.12926.

[64] G Jean-Louis et al. “Sleep estimation from wrist movement quantified by different actigraphic modalities”. In: *JOURNAL OF NEUROSCIENCE METHODS* 105.2 PG - 185-91 (2001), pp. 185–191.

[65] H Honma et al. “Motor activity rhythm in dementia with delirium”. In: *Psychiatry and clinical neurosciences* 52.2 PG - 196-8 (1998), pp. 196–198.

[66] Jesse D Cook, Michael L Prairie, and David T Plante. “Ability of the Multisensory Jawbone UP3 to Quantify and Classify Sleep in Patients With Suspected Central Disorders of Hypersomnolence: A Comparison Against Polysomnography and Actigraphy”. In: *Journal of clinical sleep medicine : JCSM : official publication of the American Academy of Sleep Medicine* 14.5 PG - 841-848 (2018), pp. 841–848. DOI: https://dx.doi.org/10.5664/jcsm.7120.

[67] M Mikulec et al. *Automatic Segmentation of Actigraphy Data Utilising Gradient Boosting Algorithm.* 2021. DOI: 10.1109/TSP52935.2021.9522650.

[68] Md Aktaruzzaman et al. “Performance comparison between wrist and chest actigraphy in combination with heart rate variability for sleep classification”. In: *Computers in biology and medicine* 89 (2017), pp. 212–221.

[69] Huaming Shen et al. “An Accurate Sleep Stages Classification Method Based on State Space Model”. In: *IEEE ACCESS* 7.PG - 125268-125279 (2019), pp. 125268–125279. DOI: 10.1109/ACCESS.2019.2939038.

[70] Daniel J Levendowski et al. “Assessment of a neck-based treatment and monitoring device for positional obstructive sleep apnea”. In: *Journal of clinical sleep medicine : JCSM : official publication of the American Academy of Sleep Medicine* 10.8 PG - 863-71 (2014), pp. 863–871. DOI: https://dx.doi.org/10.5664/jcsm.3956.

[71] I R Bell et al. “Effects of homeopathic medicines on polysomnographic sleep of young adults with histories of coffee-related insomnia”. In: *Sleep medicine* 12.5 PG - 505-511 (2011), pp. 505–511. DOI: http://dx.doi.org/10.1016/j.sleep.2010.03.013.

[72] I A Barash et al. “Nocturnal oxygen enrichment of room air at 3800 meter altitude improves sleep architecture”. In: *HIGH ALTITUDE MEDICINE & BIOLOGY* 2.4 PG - 525-33 (2001), pp. 525–533.

[73] Carmela Alcantara et al. “Sleep Disturbances and Depression in the Multi-Ethnic Study of Atherosclerosis”. In: *Sleep* 39.4 PG - 915-25 (2016), pp. 915–925. DOI: https://dx.doi.org/10.5665/sleep.5654.
